# Renal artery thrombosis in SARS-CoV-2 infection: a case report

**DOI:** 10.1186/s12882-022-02808-5

**Published:** 2022-05-06

**Authors:** Huayan Huang, Chunguang Lin, Yongdong Chen, Xiuting Wu, Miaomiao Lin, Siqi Chen, Kai Li

**Affiliations:** 1 The First People’s Hospital of Fang Cheng Gang City, Fangchenggang, 538021 Guangxi China; 2 Department of Radiology, the First Affiliated Hospital of Guangxi Medical University, Nanning, 530021 Guangxi China

**Keywords:** 2019 novel coronavirus disease, Renal artery thrombosis, Hypercoagulability

## Abstract

**Background:**

Coronavirus disease 2019 (COVID-19) is identified as the pneumonia and acute respiratory distress syndrome caused by severe acute respiratory syndrome coronavirus 2 (SARS-CoV2). The intravascular thrombotic phenomena related to the COVID-19 are emerging as an important complication that contribute to significant mortality.

**Case presentation:**

We present a 62-year-old man with severe COVID-19 and type 2 diabetes. After symptomatic and supportive treatment, the respiratory function was gradually improved. However, the patient suddenly developed abdominal pain, and the enhanced CT scan revealed renal artery thrombosis. Given the risk of surgery and the duration of the disease, clopidogrel and heparin sodium were included in the subsequent treatment. The patient recovered and remained stable upon follow-up.

**Conclusions:**

Thrombosis is at a high risk in patients with severe COVID-19 pneumonia because of hypercoagulable state, blood stasis and endothelial injury. Thrombotic events caused by hypercoagulation status secondary to vascular endothelial injury deserves our attention. Because timely anticoagulation can reduce the risk of early complications, as illustrated in this case report.

## Background

The past research shows that COVID-19 can cause arteriovenous thromboembolism in most organ systems [1–3]. Among them, what is particularly striking is the correlation between SARS-CoV-2 infection and renal artery thrombosis [1, 2]. Although the mechanism of COVID-19 complicated by renal vascular thrombosis is not yet fully understood, scholars speculate that it may be the direct pathogenic effect of SARS-CoV-2 on endothelial cells and microvascular damage, or it may be related to the hypercoagulable state of the blood [4]. Severe COVID-19 pneumonia is associated with a coagulopathic state and may increase the risk of thrombotic complications [5]. The current common thrombotic complications mainly include venous thrombosis events, pulmonary thrombosis, and myocardial infarction. However, we have noted that reports of embolization events that occur in renal arteries are rare. We present a case of renal artery thrombosis in a SARS-CoV-2-positive patient.

## Case presentation

**Fig. 1:**
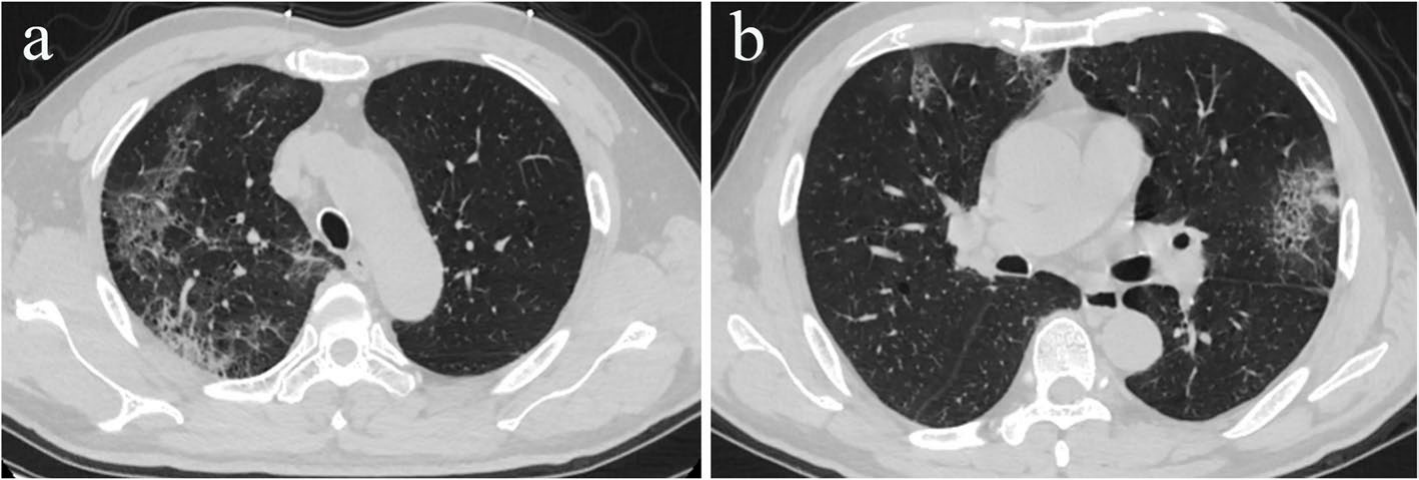
Chest CT shows ground glass-like shadows and multiple consolidations in the right upper lobe

A 62-year-old man with a history of type 2 diabetes presented with fever that started 5 days ago. Cough, fatigue, or abdominal pain were not present. At admission, the body temperature was 36.7 °C, and the patient was diagnosed with SARS-CoV-2 infection by reverse transcriptase-polymerase chain reaction (RT-PCR) assay. Vital signs on presentation revealed heart rate of 76 beats/minute, respiratory rate of 20 breaths/minute, and blood pressure of 117/65 mmHg. A chest computed tomography scan showed ground glass-like shadows multiple consolidations in the both upper lobes (Fig. 1 A to B). The initial peripheral blood sample showed that the coagulation function was normal and the LDH was 274 U/L (Table 1). The patient had no history of valvular heart disease, atrial fibrillation or urinary system disease. The electrocardiogram indicates sinus rhythm. The creatinine value is 71 μmol/L. Urinalysis results are normal. The rest of the physical examination was also unremarkable.

**Table 1:** Laboratory values on admission and the period of the onset of abdominal pain

	Admission	Period of abdominal pain	Reference range
WBC (×10^9^/L)	3.80	30.10	(4.00–10.00)
Neutrophil (×10^9^/L)	2.26	27.38	–
Lymphocyte (×10^9^/L)	0.96	0.86	–
Platelets (×10^9^/L)	161	130	150–350
C-reactive protein (mg/dL)	<5.00	140.70	0.00-10.00
Activated partial thromboplastin time (s)	33.40	26.40	25.10-35.00
D-dimer (ng/ml)	177	1008	0–243
International normalized ratio	1.13	1.00	0.80–1.20
Fibrinogen (g/L)	3.79	3.24	2-4
Fibrinogen degradation products (μg/mL)	1.09	6.14	1-5
Erythrocyte sedimentation rate (mm/h)	28	–	0-15
Antithrombin III (%)	81.80	98.60	90.3 ± 13.2
LDH(U/L)	274	576	80-285
Creatinine (μmol/L)	71	116	40-106

**Fig. 2:**
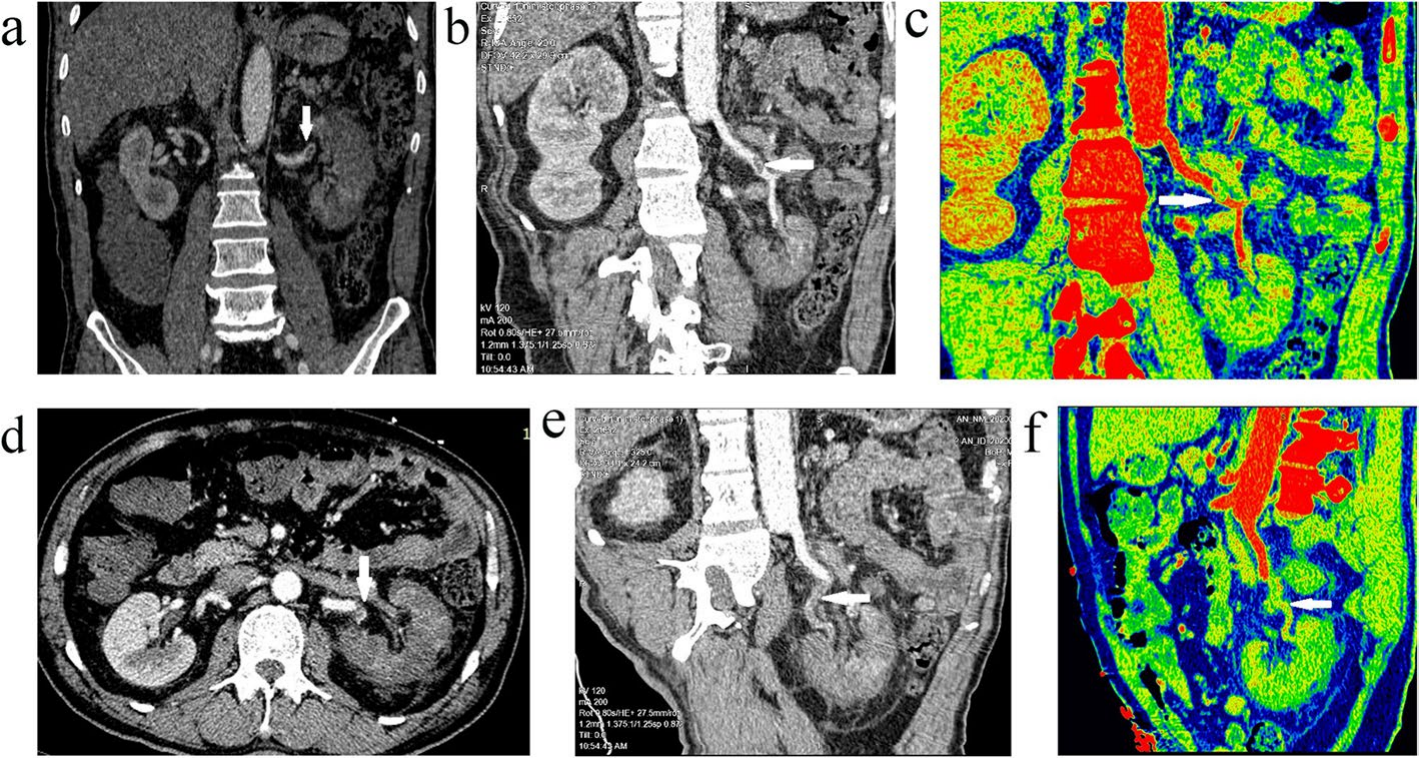
Contrast-enhanced CT indicates the filling defect of the main trunk (**a**, **b**, **c**, white arrow) and its posterior branches (**d**, **e**, **f**, white arrow) of the left renal artery

According to the fifth edition of Chinese New Coronavirus (2019-nCoV) Infection Treatment Plan Guide [6], the coronavirus pneumonia diagnosed was classified as mild type. He agreed to be given routine antiviral treatment and glycemic management of diabetes. Three days later, the patient developed shortness of breath and the symptom gradually worsened. The blood gas analysis showed an oxygenation index result of 172. After a regimen of 4-day course of methylprednisolone and ceftriaxone, his physical condition showed a significant improvement. On day 12 of hospitalization, the patient reported sudden-onset severe pain in the left waist. Abdominal and urinary color doppler ultrasound at that time showed no abnormalities. Two days later, laboratory tests displayed significantly elevated d-dimer level of 1008 ng/ml, creatinine level of 116 umol/L, and LDH level of 576 U/L. Urine routine showed urinary occult blood 2 + and protein 2 +. Table 1 shows the results of the second peripheral blood test. The enhanced CT scan revealed the left renal artery embolism and threatening ischemia (Fig. 2 A to F). The electrocardiogram of this patient showed sinus rhythm, and there was no history of cardiac valvular disease and atrial fibrillation, which did not support cardiogenic embolism, so it was diagnosed as renal artery thrombosis, resulting in acute renal artery embolism. Although the patient did not have obvious contraindications to thrombolysis, thrombosis occurred in the renal artery branch, interventional procedures were difficult and risky. The patient was diagnosed as renal artery thrombosis 48 hours after the onset of symptoms, and thrombolytic reperfusion therapy may not be beneficial. The patient had not received any anticoagulant therapy before. Considering that the process of renal artery thrombosis involves platelet activation and aggregation, clopidogrel antiplatelet aggregation therapy (clopidogrel 300 mg with 75 mg daily) and nadroparin calcium (3800 IU/q12h) anticoagulant therapy were given. Three days later, clopidogrel combined with rivaroxaban was used to maintenance therapy. The patient was discharged from the hospital after 1 week of anticoagulation therapy, with disappearance of lumbago, negative lumbar percussion pain, reexamination of D-dimer and renal function, and normal urinary routine. After that, the patient continued to receive the above anticoagulant regimen for 3 months. After 3 months, the patient returned to the hospital for reexamination, indicating that the renal function was normal.

## Discussion and conclusion

In this case, renal artery thrombosis suddenly occurred during the treatment of COVID-19 and subsequently led to left renal threatening ischemia. The laboratory test indicating thrombotic complications was elevated D-dimer. Previous studies have shown that elevated D-dimer was a risk factor for death in patients with SARS-CoV-2 infection, especially in elderly patients [7, 8]. The hypercoagulation status of COVID-19 and the subsequent series of vascular complications are a vital topic that is currently emerging [9]. Previous reports have suggested that patients with renal artery thrombosis were patients with atrial fibrillation or had a history of renal artery stenting. Interestingly, the patient in this case had no history of atrial fibrillation or renal artery surgery, and the occurrence of renal artery thrombosis suggested that it was related to vascular damage and coagulation function changes in novel Coronavirus. This has implications for future renal injury complications from COVID-19 [10–12]. The question of anticoagulant therapy at prophylaxis dose or even higher was subsequently raised [13]. It has been suggested that immune-mediated reaction or direct viral infection of the endothelium will lead to recruitment of immune cells, which may develop into extensive endothelial dysfunction [14]. Angiotensin converting enzyme 2 (ACE2) receptors are expressed in organs including lung, heart, kidney and intestine. Vascular endothelial cells also express angiotensin converting enzyme 2 receptor. And the virus can directly infect endothelial cells by converting enzyme 2 and cause diffuse endothelial inflammation [15]. SARS-CoV-2 infection can develop into acute respiratory distress syndrome. Similar to other viral infections, early cytokine storms are caused by overproduction of response proinflammatory cytokines including tumor necrosis factor, interleukin-6 and interleukin-1 β, resulting in an increased risk of multiorgan failure and vascular hyperpermeability [16]. The main function of thrombin in the immune response is to promote the formation of clots by activating platelets and converting fibrinogen to fibrin. However, it is worth noting that the cellular effect of thrombin, mainly par-1 (proteinase-activated receptors), can further enhance inflammation [17]. Protein C system, tissue factor pathway inhibitor and antithrombin are defective during inflammation, which impairs the coagulant–anticoagulant balance and leads to the formation of microthrombus [18]. The hypercoagulation status of blood caused by the infection of SARS-CoV-2 can lead to a variety of intravascular thrombotic phenomena, ranging from limited venous and arterial thrombosis to fatal disseminated intravascular coagulation.

Thrombotic events caused by hypercoagulation status secondary to vascular endothelial injury deserves our attention. Because timely anticoagulation can reduce the risk of early complications.

## Data Availability

If required, the relevant material can be provided by corresponding author on reasonable request.

## Abbreviations

COVID-19: Coronavirus disease 2019
SARS-CoV2: severe acute respiratory syndrome coronavirus 2
